# Sedentary Lifestyle, Physical Activity, and Healthy Digital Media Use of Filipino Adolescents: Review and Policy Insights

**DOI:** 10.1002/hsr2.71012

**Published:** 2025-06-30

**Authors:** Danilo V. Rogayan, Edna M. Padre

**Affiliations:** ^1^ College of Teacher Education President Ramon Magsaysay State University Iba Zambales Philippines; ^2^ Graduate School President Ramon Magsaysay State University Iba Zambales Philippines; ^3^ College of Arts & Sciences President Ramon Magsaysay State University Iba Zambales Philippines

Dear Editor,

Recent findings on leisure sedentary behaviors (LSBs) and physical activity (PA) underscore the importance of differentiating the types and intensities of these behaviors in assessing health outcomes, such as gastroesophageal reflux disease (GERD). While leisure television watching and self‐reported moderate PA were found to increase GERD risk, other behaviors like leisure computer use and objectively measured PA (via accelerometer data > 425 milligravities) showed protective associations [1]. These insights prompt a closer look at Filipino adolescents, who are among the most sedentary youth globally.

In 2019, the World Health Organization (WHO) reported that 93.4% of Filipino adolescents were physically inactive—ranking the Philippines second only to South Korea in global adolescent inactivity [2]. Complementary findings from the Active Healthy Kids Global Alliance (AHKGA) in their Youth Physical Activity Report Cards further revealed that only 15.4% of Filipino adolescents met global PA guidelines [3]. These sobering statistics highlight a pervasive issue that has persisted even after pandemic lockdowns, pointing to the urgent need for multilevel intervention and sustained commitment from all sectors of society.

The 2022 Philippine Report Card on Physical Activity for Children and Adolescents offers a holistic overview of the PA landscape in the country, assessing behaviors, influential settings, and existing policy supports [4]. This document is vital in amplifying awareness among educators, parents, and policymakers. Notably, ten government policies related to PA were evaluated in this report, affirming the state's potential to effect change through strategic legislation and enforcement [5]. However, policy presence alone is insufficient. Implementation fidelity and ongoing evaluation are essential to translate these measures into meaningful behavior change among Filipino youth.

In tandem with physical inactivity, digital media consumption has dramatically increased. A 2021 survey reported that 97% of Filipino adolescents aged 16–17 engage in social media weekly, often accessing content from the comfort of their homes [6]. While technology can serve educational and social functions, its excessive and unregulated use is frequently linked to sedentary habits and poor health outcomes. Therefore, promoting healthy digital media behaviors is equally critical.

A balanced approach is needed—one that not only reduces sedentary screen time but also leverages technology to promote physical health. Policy efforts must include curriculum‐based physical education, active classroom breaks, and the promotion of community‐based PA programs. Simultaneously, digital literacy campaigns should empower adolescents and their parents to manage screen time wisely. Encouraging the use of fitness applications and online exercise programs can create opportunities to merge digital engagement with active lifestyles.

Combating sedentary behavior and promoting healthy digital media use among Filipino adolescents demands a comprehensive, multisectoral strategy. Government agencies, educational institutions, communities, and families must collaborate to create environments that support active, balanced, and health‐promoting lifestyles in an increasingly digital world.

## Author Contributions

**Danilo V. Rogayan:** conceptualization, writing – original draft, writing – review and editing. **Edna M. Padre:** writing – review and editing.

## Ethics Statement

Ethical standards are followed in the research.

## Conflicts of Interest

The authors declare no conflicts of interest.

## Transparency Statement

The lead author, Danilo V. Rogayan Jr., affirms that this manuscript is an honest, accurate, and transparent account of the study being reported; that no important aspects of the study have been omitted; and that any discrepancies from the study as planned (and, if relevant, registered) have been explained. All authors have read and approved the final version of the manuscript, the corresponding author had full access to all of the data in this study and takes complete responsibility for the integrity of the data and the accuracy of the data analysis.

## Data Availability

All the data analyzed in this review were obtained from previously published articles, which are cited throughout the text. These references can be accessed through their respective publishers. For detailed information on the sources, please refer to the reference section of this manuscript. No new or unpublished data were utilized or generated in the preparation of this review.
